# Annotations

**Published:** 1892-03-26

**Authors:** 


### Annotations.

**Gold against A cohol.**

"There is one vice, or disease, whatever it be—where doctors differ it is not for laymen to decide—which has of late years been steadily increasing among the educated classes of this country," says an American writer, and that is drunkenness. The cure? Ay, there's the rub! But he goes on to tell of a cure, or at least, to use his own cautious phrase, "a marvellously clever imitation of a cure," discovered by Dr. Leslie E. Keeley. There are three "Keeley Institutes" in the State of New York where inebriates can go for a three weeks' course of inoculation with bi-chloride of gold, the supposed specific for the craving for stimulants—alcohol, chloral, morphia, or any of the other drugs which a weak will and shattered nerves demand. Four times a day do the patients submit to a hypodermic injection of the bi-chloride, and the rest of the treatment consists simply in leading a quiet and reasonable life. The use of tobacco is forbidden during the quarter of an hour before, and that after the injection, but the rest of the day the patient may smoke anything he likes except a cigarette. "Cigarette smoking and gambling will be punished by instant dismissal," says the fifth rule of the institute. Patients are asked also to take no alcohol except what they get at the institute, not because this is "doctored," but, on the contrary, because it is pure six-year-old whisky. But it is said that after the third day the patients rarely feel any desire for a stimulant, and the offer of "as much as you like" contains no temptation. An internal mixture, containing a variable dose of the bi-chloride, specially graduated to suit the needs of the individual patient is administered, and this, with the injection, is said to restore the stomach to the state it was in before the owner learned to drink, and thus frees him from the temptation to spur on the jaded organ with a fresh dose of the poison that is killing him. It is not claimed that the bi-chloride of gold is an infallible cure for the alcohol or morphia habit, but if it is true, as is alleged, that ninety-five per cent. of those who have "graduated," as they term it from these institutes, can resist the old temptation, the cure is worth a more serious trial than we are always willing to give to things American.

**Doctors ask for a Little Reciprocity.**

The words "Medical charity" are amongst the most familiar of all the expressions that are found in the current newspapers of every class. Whether willingly or by compulsion it matters little, the thirty thousand medical men of this country give more money, or money equivalent, in charity every year than any other thirty thousand persons of any class in the population. We are not saying that doctors are more charitable than other people, nor do we believe that they are, but the nature of their calling is such as to compel, practically, every general practitioner to give at least a seventh or an eighth of his money-earning power to the public for nothing during the whole of his working life. This is a most serious drag upon doctors, many of whom have painfully small incomes; but being on the whole well-conditioned Britons, they recognise that it is inevitable, and they do not complain. But this service, which they render so ungrudgingly, does, we think, entitle them to a little special consideration at the hands of the public when members of the profession are themselves driven by stress of circumstances to seek charitable help rather than to give it. The medical profession as a whole supports fifty aged pensioners who have fallen wounded in its ranks at a home in connection with Epsom College. It also maintains fifty foundation scholars, the orphan sons of deceased medical practitioners who have died so young as to have been unable to make adequate provision for their families. The managers of these charitable schemes now find it incumbent upon them to provide education and maintenance for a good many more than fifty foundation scholars. They need in short to add to the school buildings all the accommodation occupied by the fifty broken down medical pensioners. What they propose to do in this emergency is we think the very wisest and kindest thing they could do. They propose to give a little increase to the pensions of the aged medical men in lieu of the rooms they are now provided with at Epsom College, and to let them live where they please. The poor old gentlemen are delighted with the idea of regaining what they consider their "lost liberty." The scheme is eminently considerate and humane, but it will cost something over £30,000. Towards this sum the more prosperous members of the medical profession will contribute a good deal. But it must be remembered that the whole support both of the fifty aged pensioners and of the fifty foundation scholars has practically been contributed by the profession. To ask them for more is therefore to lay weight upon a burden which is already almost greater than they can bear. The Lord Mayor of London, as the mouth-piece and representative of all the charities, has generously come forward in these urgent circumstances. He will carry the appeal from the small and comparatively poor body of the medical profession to the rich and numerous public. His lordship will take the chair at the Biennial Festival of this medical benevolent institution on April 5th, and a strong and united effort will be made to raise the needed £30,000. The editors of the *Lancet* have challenged the charitable public to do this reciprocal kindness to the medical profession by offering to give a thousand pounds on condition that the whole thirty thousand is collected. We make a very urgent appeal on this behalf to the many thousand readers of the Hospital. We think that they alone might have the honour of contributing ten of the thirty thousand pounds required. If the readers of the *Lancet* and of the *British Medical Journal* would each contribute other ten thousand pounds the thing would be done. Dr. Charles Holman, the Honorary Treasurer, or Mr. J. B. Lamb, the Secretary, will be glad to hear from any warm-hearted friends of aged doctors, and doctors' orphans, at 37, Soho-square, W.

**Dirty Larders.**

Dr. Ballard, in the annual report of the Medical Department of the Local Government Board just issued, records fourteen instances in which specific diseases had been originated or spread by articles of food. It is commonly believed that meats of various kinds, such as fish, fowl, beef, mutton, and the like, become poisonous by their own decomposition when kept too long. And this common belief is well founded. But Dr. Ballard considers that at least several of the cases he mentioned did not originate in decomposing meat, but in poisonous and deleterious matters which were to be found in the pantries and larders where the meat had been kept, and which had been deposited upon it from the surrounding atmosphere, and the generally dirty environment. The present writer has seen several cases of severe gastric poisoning which he considered due to the impure atmospheric conditions of larders in which meat had been kept. It does not seem to be generally understood that meat, and other articles that are usually stored in pantries and larders, give off exhalations which settle down upon walls, floors, ceilings, and any tables or vessels that may be present, and also at the same time charge the atmosphere of the larder or pantry with concentrated poison. Such, however, are the undoubted facts. The only way in which meat and other things can be preserved in a wholesome condition, is that of keeping floors, walls, and ceilings, as well as tables and vessels scrupulously clean, to whitewash with quicklime, or to have the walls lined with glazed tiles, and to have the freest possible circulation of pure atmospheric air through every part of the larder or pantry both day and night. Dr. Ballard is of opinion that much more care should be given than is now commonly the case to secure the most cleanly and wholesome conditions for pantries and larders, as well as for places where meat is being manipulated in the course of its preparation for sale. These statements seem to be of a purely elementary character; everybody thinks he knows all about them. But in sober earnest people do not really know, for if they did, they would surely not be so foolish as to run such risks of wholesale poisoning as they now indulge in. If all people, instead of reading so much with a view to the gaining of so much more knowledge, would try to carry out in practice the despised elementary knowledge they already possess, they would show far more real intellectual capacity, and be both healthier and happier. It is with science as Mr. Matthew Arnold says it is with religion, conduct, and not the mere acquisition of knowledge is three-fourths of the battle and the victory.

